# Prevalence and Prognostic Value of Psychological Stress Events in Patients with First Myocardial Infarction—Long-Term Follow-Up Study

**DOI:** 10.3390/jcm10163562

**Published:** 2021-08-13

**Authors:** Andrea Jaensch, Ben Schöttker, Roman Schmucker, Wolfgang Koenig, Hermann Brenner, Dietrich Rothenbacher

**Affiliations:** 1 Institute of Epidemiology and Medical Biometry, Ulm University, 89081 Ulm, Germany; andrea.jaensch@uni-ulm.de (A.J.); koenig@dhm.mhn.de (W.K.); 2 Division of Clinical Epidemiology and Ageing Research, German Cancer Research Center (DKFZ), 69120 Heidelberg, Germany; b.schoettker@Dkfz-Heidelberg.de (B.S.); h.brenner@Dkfz-Heidelberg.de (H.B.); 3 Network Ageing Research, University of Heidelberg, 69115 Heidelberg, Germany; 4 Klinik Schwabenland, 88316 Isny-Neutrauchburg, Germany; Roman.Schmucker@wz-kliniken.de; 5 Deutsches Herzzentrum München, Technische Universität München, 80636 Munich, Germany; 6 German Centre for Cardiovascular Research (DZHK), Partner Site Munich Heart Alliance, 80636 Munich, Germany

**Keywords:** coronary heart disease, myocardial infarction, psychological stress events, depression, anxiety

## Abstract

While there is good evidence that symptoms of depression determine prognosis of patients with coronary heart disease (CHD), the role of psychological stress is less clear. We evaluated the prognostic value of stressful events in patients with initial myocardial infarction (MI) with respect to subsequent cardiovascular events. The KAROLA-study included patients with CHD who participated in an in-patient rehabilitation program. A total of 577 patients with initial MI were included and self-reported psychological stressful events before their MI was assessed by a structured questionnaire. Hazard ratios were used to evaluate the long-term association of stressful events with secondary cardiovascular events. Additionally, associations of stressful events with depression, anxiety and other cardiovascular risk factors were investigated. Unusual stress at work (26.5%) and sleep disorder (23.4%) were the most frequently reported stressful events that occurred in the last 4 weeks before MI. However, only death of a family member showed a statistically significant increase in risk for subsequent cardiovascular events (HR: 1.59; 95%-CI: 1.01–2.50) and this result was not corrected for multiple testing. Notably, we found higher symptom scores of anxiety and depression associated with all single stressful event items. In conclusion, we found no clear patterns that psychological stressful events before MI would increase the long-term risk of subsequent adverse CHD events directly. However, we saw increased symptom scores of anxiety and depression in persons with stressful events.

## 1. Introduction

Patients with coronary heart disease (CHD) often report symptoms of depression and anxiety [1,2]. There is good evidence that especially symptoms of depression determine the prognosis of patients with CHD [3]. As a consequence, a higher priority is given to psychosocial factors in recent cardiologic guidelines, and mental health risk factors such as depression are established risk and prognostic factors for CHD. Yet they seem to be under-recognized in their importance, and are often not incorporated in secondary prevention strategies in clinical practice, especially during follow-up care [4].

However, besides mood disorders such as depression and anxiety, other negative psychological stress factors receive less attention, and evidence from studies is scarce. Little data exists about the prevalence and the role of psychological stress and trauma as triggers of acute events and even less about their role as prognostic factors for long-term outcomes. These psychological risk factors and psychological stress are probably also associated with established cardiovascular risk factors and associated adverse endpoints. However, key questions about directionality and possible causal relationships are still open [5].

The objective of this study was to evaluate the prevalence and the prognostic value of psychological stress events in patients with first myocardial infarction (MI) with respect to subsequent cardiovascular events.

## 2. Materials and Methods

### 2.1. Study Population and Design

KAROLA, a prospective cohort study, included all patients with incident CHD (International Classification of Diseases, 9th Rev. pos. 410–414) who participated in an in-patient rehabilitation program between January 1999 and May 2000 in one of two rehabilitation clinics in Germany (Schwabenland-Klinik, Isny, and Klinik am Südpark, Bad Nauheim). The age of the patients included was between 30 and 70 years (details of the overall CHD cohort in [3]). In Germany, all patients with acute MI or coronary artery revascularization (coronary artery bypass grafting (CABG), or percutaneous coronary interventions (PCI)) are offered an in-hospital rehabilitation program after discharge from the acute care hospital. This program lasts about three weeks and in KAROLA, only patients who were admitted within three weeks after their first acute event were included. During the recruitment period, 58% (*n* = 1206) of all eligible patients agreed to participate. The study was approved by the Ethics Boards of the Universities of Ulm (No. 186/98) and Heidelberg and of the Physicians’ chamber of the States of Baden-Württemberg and Hessen.

### 2.2. Data Collection and Assessment of Psychological Stress Events 

All patients completed a standardized questionnaire at the beginning of the in-patient rehabilitation program, containing sociodemographic information, medical history and the German version of the Hospital Anxiety and Depression Scale (HADS) [6,7]. Patients after MI also received a structured questionnaire with further information on the MI event and its circumstances. Only these patients were included in this study (*n* = 577). Psychological stress events were asked as follows: “Did one of the following events happen in the time before infarction, which was very stressful for you?”. Possible answers were “No”, “Yes, within 24 h before infarction”, “Yes, within 4 weeks before infarction” and “Yes, more than 4 weeks before infarction”. The following events were recorded one after another: “Death of family member”, “Death of friend”, “Personal disease”, “Unusual stress at work”, “Mental excitement/dispute”, “Sleep disorders”, “Changes at workplace” and “Changes in partner relationship”. Besides evaluating the single items, a score-variable was also built by summing up the events.

### 2.3. Follow-Up and Evaluation of Cardiovascular Disease Events

For follow-up, all patients and their primary care physicians were contacted simultaneously by mail one, three, four and a half, six, eight, ten and fifteen years after discharge from in-patient rehabilitation. Patients completed a standardized questionnaire and primary care physicians were asked about adverse cardiovascular disease events and treatment since discharge from the rehabilitation clinic. In case of death during follow-up, the death certificate, with the date and main cause of death, was obtained from local Public Health departments. The main cause of death was coded according to the International Classification of Disease. A subsequent adverse cardiovascular disease event (CVD) was defined as: CVD as main cause of death (ICD-9 pos. 390–459; ICD-10 pos. I0-I99 and R57.0) or diagnosed non-fatal MI, or ischemic cerebrovascular event (TIA or stroke) as reported by the primary care physicians.

### 2.4. Statistical Analysis

After describing the study population with respect to various sociodemographic and medical characteristics, the prevalence of stressful events occurring within 4 weeks before MI was determined. To investigate the association of stressful events with subsequent non-fatal and fatal secondary CVD events, we fitted a Cox proportional hazard model for 15 years of follow-up (hazard ratio (HR) and its 95% confidence interval (CI)). We also considered six years of follow-up, assuming that the association might be stronger during shorter follow-up periods, as the influence of specific point-in-time related stressful events might decrease over time. The basic model was adjusted for age and sex. Additional established potential confounders were considered in the second model: body mass index (BMI), education (<10 years vs. ≥10 years), rehabilitation clinic, smoking status (never, current, former-smoker), history of diabetes mellitus, left ventricular function (no or only little impairment, modest or severe impairment) and high-density lipoprotein cholesterol.

We also investigated the association of psychological stressful events with suspected cardiovascular risk factors such as body mass index (BMI), diastolic and systolic resting blood pressure, lipid parameters, cotinine level in serum, C-reactive protein, NT-proBNP, hs-cTnT, and symptom scores of depression and anxiety. Age- and sex-adjusted mean values were calculated and compared according to the presence or absence of each stressful event by means of Generalized Linear Regression Models. All statistical procedures were performed with the SAS statistical software package (release 9.4 SAS Institute Inc., Cary, NC, USA).

## 3. Results

The mean age of the 577 included patients with MI was 57.3 years at baseline and 84% of them were male. Further characteristics are displayed in Table 1. 

As depicted in Table 2, about one third of the patients reported unusual stress at work (33.0%) or sleep disorders (28.0%) before MI. Mental excitement or dispute (15.9%) or personal disease (14.7%) were also common. When only events within four weeks before MI were considered, unusual stress at work (26.5%), sleep disorders (23.4%), and mental excitement or dispute (11.8%) remained the most commonly reported events.

The risk for subsequent secondary CVD events (*n* = 171) during 15 years of follow-up increased for some of the stressful events, but none were statistically significant except death of a family member in the fully adjusted model (HR: 1.59; 95%-CI: 1.01–2.50) (Table 3). In addition, a summary score of stressful events was not associated with adverse CVD outcomes in our multivariable model. 

The association of stressful events before MI with age- and gender adjusted symptom scores of depression and anxiety and other established cardiovascular risk factors were evaluated at baseline. We found higher symptom scores of anxiety and depression associated with all single stressful event items. In detail, a statistically significant higher depression score was found among study participants who reported personal disease, temporarily unusual stress at work, sleep disorders, and changes in partner relationship. Symptoms of anxiety were significantly associated with personal disease, unusual stress at work, sleep disorders, changes at workplace and changes in partner relationship (Table 4). Most of the other well-established cardiovascular risk factors did not differ substantially between presence or absence of a specific stressful event and showed no consistent patterns with stressful events (Table S1 in the Supplementary Materials).

## 4. Discussion

In this long-term cohort study including 577 patients with initial MI, besides an association of the stressful event “death of a family member”, we found no consistent indication that psychologically stressful events before MI increased the long-term risk of subsequent adverse CHD events in a direct manner. However, we found an accumulation of increased symptom scores of anxiety and depression clustering in patients reporting stressful events. Nevertheless, the temporal sequence, especially regarding symptoms of anxiety and depression, the latter being recognized as a well-established prognostic risk factor, remains unclear. We also cannot discriminate causes from consequences of stress.

The prevalence of stressful events in our study varied considerably. Whereas changes in partner relationship were reported in only 3.4% of patients (0.5% during past four weeks), stress at work, with a prevalence of 33.0%, and sleep disorders, with a prevalence of 28.0% (in past 4 weeks 26.5% and 23.4%, respectively), were very common. Although there is much evidence that perceived work stress is associated with an increased risk of incident CHD, evidence investigating the association of work stress with prognosis in patients with CHD is limited, but points to an increased risk for recurrent CVD events [8]. Kivimäki et al. [9] described that stress in adulthood may rather act as a trigger of acute CVD in high-risk populations for CVD, but there is also evidence that several stressors, such as perceived work stress, perceived stress and persistent psychological distress, are associated with adverse events in adults with existing CVD [9]. Work stress as measured by the well-established effort–reward imbalance instrument (i.e., high demand and low control or high effort and low reward) was associated with a 65% increase for recurrent CVD events in a meta-analysis done by Li et al. [8]. Perceived stress levels as measured by the validated perceived stress scale occurred with a 42% increased risk for 2-year mortality in a large cohort of patients with acute MI [10]. Although perceived stress was highly associated with depressive symptoms as in our study, the association of stress persisted after adjustment for depressive symptoms in their study.

The evidence base for the association of anxiety and depression, especially for symptoms of depression, and their adverse relationship with prognosis in patients with CVD is better. It is likely that persistent psychological distress over time, instead of one-point measurements, should be considered. This was done in a study with the General Health Questionnaire over 4 years in 950 patients with a history of acute MI to demonstrate an association with long-term adverse outcomes [11]. 

In a previous analysis of the KAROLA-study, we had found a relevant prognostic association of anxiety and depression with further CHD events [3]. Stress at work and in family life, hostility, depression, anxiety and other mental disorders are considered as psychological risk factors in current ESC guidelines which contribute to the risk of developing incident CVD and also worsen the prognosis [4]. Screening is recommended at initial assessment and it appears that a long-term perspective should be implemented in the care of patients with CHD. Looking at the age- and sex-adjusted mean values, we clearly saw higher values of the anxiety and depression scores for patients with a stressful event. Furthermore, patients with one event were more likely to have other events. This indicates that a certain vulnerability or awareness of stress is more present in patients with a history of depression or anxiety. Psychological aspects such as purpose of life may also be important, as individuals with a high purpose of life may perceive stressors as less difficult, and might be less likely to react with unhealthy behaviour (i.e., unhealthy diet, smoking, less adherence to medication, etc.) [12]. 

The associations between stressful events and adverse cardiovascular events are complex. Interrelation with well-established behavioural and physiological risk factors such as smoking, unhealthy food choices, increased blood pressure, but also with symptoms of anxiety and depression, could all play a role. On the physiological levels, alterations in the autonomic nervous system and changes in other endocrine markers which are also related to hemostatic and pro-inflammatory changes, are discussed as possible links between stress, anxiety and depression and adverse cardiovascular outcomes, including death [4]. However, stressful events and other mental health disorders should be addressed and treated irrespective of a possible link with CHD prognosis.

This study has strengths and limitations. Overall, we investigated different domains of stressful events and investigated their association with established cardiovascular risk markers at baseline, and with mid-term as well as long-term adverse CVD events. Strengths include the long-term follow-up and the availability of well-established biomarkers. Furthermore, depression and anxiety were investigated with a standardized and validated questionnaire, which is established and worked well in patients with cardiac disease. Limitations include the lack of qualitative information to include additional sources of stressful events, the lack of validation of questions about psychological stressful events before MI and the one-time measurement. Moreover, as questions were asked after the events, recall and response bias might have been an issue. We did not correct for multiple testing (e.g., by means of Bonferroni-correction) and the study had a limited power due to the sample size. In addition, personality type and coping strategies were not assessed in patients with MI, as both might make an important difference. This notion is supported by the accumulation of stressful events within persons reporting such events and the association of stressful events with higher symptoms of anxiety and depression scores.

## 5. Conclusions

In conclusion, in patients with CHD, self-reported, different psychological stressful events before MI were not consistently associated with risk for subsequent CHD events. However, we found an accumulation of increased symptom scores of anxiety and depression clustering in patients reporting stressful events, suggesting that it might be useful to screen for mood disorders in patients with a history of stressful events during follow-up care and treat affected patients accordingly.

## Figures and Tables

**Table 1:** Descriptive table of the study population with myocardial infarction at baseline (*n* = 577).

Characteristics at Baseline	
Age (years) (µ, SD ^#^)	57.3 (8.6)
Men, *n* (%)	485 (84.1%)
Length of follow-up, years *	13.0 (6.7; 14.8)
School education < 10 years, *n* (%)	342 (59.3%)
Body mass index (kg/m^2^), (µ, SD)	27.1 (3.3)
Smoking status, *n* (%)	
Never	177 (30.7%)
Former	365 (63.3%)
Current	35 (6.1%)
History of diabetes, *n* (%)	91 (15.8%)
History of hypertension, *n* (%)	293 (51.2%)
History of heart failure, *n* (%)	46 (8.3%)
Clinical score (angiographic evaluation), *n* (%)	
1 vessel disease	209 (36.2%)
2 vessel disease	165 (28.6%)
3 vessel disease	152 (26.3%)
Unknown	37 (6.4%)
Left ventricular function, *n* (%)	
Unknown	41 (7.1%)
Little/no	392 (67.9%)
Modest/severe	144 (25.0%)
Percutaneous coronary intervention, *n* (%)	316 (55.1%)
Coronary artery bypass graft, *n* (%)	106 (18.5%)
HDL-cholesterol ^§^ (mg/dL) (µ, SD)	40.3 (11.0)
C-reactive protein (mg/L) *	2.35 (0.97; 5.87)
NT-proBNP (ng/L) *	606.3 (291.6; 1193.0)
hs-Troponin T (ng/L) *	12.60 (8.19; 21.40)

* Median (Interquartile range). # Standard deviation. § High-density lipoprotein cholesterol.

**Table 2:** Number and percentage of persons with stressful events before myocardial infarction happened any time or within 4 weeks before infarction.

	Any Time *n* (%)	Up to 4 Weeks Before Infarction *n* (%)
Death of family member	60 (10.6%)	13 (2.3%)
Death of friend	27 (4.8%)	10 (1.8%)
Personal disease	83 (14.7%)	37 (6.5%)
Unusual stress at work	187 (33.0%)	150 (26.5%)
Mental excitement/dispute	89 (15.9%)	66 (11.8%)
Sleep disorders	159 (28.0%)	133 (23.4%)
Changes at workplace	43 (7.7%)	9 (1.6%)
Changes in partner relationship	19 (3.4%)	3 (0.5%)

**Table 3:** Association of reporting stressful events before MI with fatal and non-fatal CVD events during 15 years of follow-up (FU).

	*n* Events (%)	FU-Time in Years (Median)	HR (95% CI) Adjusted for Age and Sex	HR (95% CI) Adjusted for Multiple Covariates *
Death of family member	22 (36.7%)	12.2	1.44 (0.92–2.26)	1.59 (1.01–2.50)
Death of friend	9 (33.3%)	13.7	0.98 (0.50–1.92)	0.97 (0.49–1.90)
Personal disease	29 (34.9%)	13.0	1.22 (0.82–1.82)	1.20 (0.80–1.79)
Unusual stress at work	41 (21.9%)	13.8	0.69 (0.48–1.00)	0.77 (0.53–1.12)
Mental excitement/dispute	23 (25.8%)	13.1	0.97 (0.62–1.51)	0.98 (0.62–1.53)
Sleep disorders	51 (32.1%)	13.0	1.11 (0.80–1.55)	1.14 (0.82–1.60)
Changes at workplace	11 (25.6%)	13.0	1.14 (0.61–2.13)	1.19 (0.63–2.24)
Changes in partner relationship	7 (36.8%)	11.5	1.65 (0.77–3.53)	1.92 (0.88–4.20)
Score of stressful events	104 (28.0%) ^#^	13.0 ^#^	1.02 (0.90–1.16)	1.05 (0.92–1.20)

* Age, sex, BMI, education, rehabilitation clinic, smoking status, history of diabetes mellitus, left ventricular function and high-density lipoprotein cholesterol. ^#^ Score > 0.

**Table 4:** Association of stressful events before MI with age- and sex-adjusted mean values of symptom scores of depression and anxiety.

	Depression Score	Anxiety Score
Death of family member		
Yes	5.65	6.74
No	4.78	5.99
*p*-value	0.089	0.18
Death of friend		
Yes	5.73	6.23
No	4.85	6.08
*p*-value	0.22	0.85
Personal disease		
Yes	5.67	7.10
No	4.72	5.87
*p*-value	0.033	0.010
Unusual stress at work		
Yes	5.33	6.88
No	4.64	5.65
*p*-value	0.059	0.002
Mental excitement/dispute		
Yes	5.32	6.75
No	4.80	5.95
*p*-value	0.24	0.088
Sleep disorder		
Yes	5.54	6.76
No	4.48	5.66
*p*-value	0.002	0.003
Changes at workplace		
Yes	5.81	7.46
No	4.71	5.85
*p*-value	0.074	0.016
Changes in partner relationship		
Yes	8.89	9.10
No	4.69	5.92
*p*-value	<0.001	0.001

## Data Availability

The datasets used and/or analysed during the current study are available from the corresponding author on reasonable request.

## Supplementary Materials

The following are available online at https://www.mdpi.com/article/10.3390/jcm10163562/s1, Table S1: Association of stressful events before MI with age- and sex-adjusted mean values or concentrations (at the end of rehabilitation) of cardiovascular risk factors and biomarkers.
