# Supplementary material for: Prevalence and Prognostic Value of Psychological Stress Events in Patients with First Myocardial Infarction—Long-Term Follow-Up Study
Source: J Clin Med. 2021 Aug 13;10(16):3562. doi: 10.3390/jcm10163562 (PMC8397004; doi:10.3390/jcm10163562)
Supplement: Supplementary file 1 [file jcm-10-03562-s001.zip › jcm-1313778-SI.pdf]

Table S1. Association of stressful events before MI with age- and sex-adjusted mean values or concentrations (at the end of rehabilitation) of cardiovascular risk factors and biomarkers.

| | BMI[*] (kg/$m^2$) | DBP[#] (mmHg) | SBP[§] (mmHg) | Total cholesterol (mg/dl) | HDL cholesterol (mg/dl) | Triglycerides (mg/dl) | Cotinine[$] (µg/l) | CRP[&] (mg/l) | BNP[+] (ng/l) | Hs- troponin (ng/l) |
|---|---|---|---|---|---|---|---|---|---|---|
| Death of family member | | | | | | | | | | |
| Yes | 27.77 | 73.40 | 120.18 | 174.87 | 40.86 | 149.82 | 92.76 | 4.96 | 969.43 | 12.68 |
| No | 27.09 | 72.24 | 115.96 | 171.85 | 42.19 | 151.57 | 91.75 | 5.10 | 1004.22 | 16.62 |
| p-value | 0.14 | 0.36 | 0.040 | 0.52 | 0.38 | 0.87 | 0.96 | 0.93 | 0.92 | 0.34 |
| Death of friend | | | | | | | | | | |
| Yes | 27.47 | 68.39 | 110.42 | 168.09 | 40.66 | 167.28 | 81.93 | 3.91 | 1454.36 | 17.83 |
| No | 27.15 | 72.57 | 116.73 | 172.40 | 42.08 | 150.41 | 92.34 | 5.05 | 981.01 | 16.12 |
| p-value | 0.61 | 0.019 | 0.028 | 0.51 | 0.51 | 0.25 | 0.71 | 0.58 | 0.30 | 0.77 |
| Personal disease | | | | | | | | | | |
| Yes | 26.69 | 72.61 | 115.84 | 172.23 | 43.14 | 135.03 | 77.26 | 3.88 | 1128.94 | 15.15 |
| No | 27.27 | 72.28 | 116.50 | 172.22 | 41.80 | 154.03 | 94.90 | 5.34 | 973.80 | 16.41 |
| p-value | 0.15 | 0.77 | 0.71 | 1.00 | 0.32 | 0.038 | 0.31 | 0.28 | 0.59 | 0.73 |
| Unusual stress at work | | | | | | | | | | |
| Yes | 26.89 | 71.66 | 115.50 | 173.89 | 41.45 | 148.70 | 98.27 | 4.76 | 685.35 | 12.25 |
| No | 27.32 | 72.80 | 116.90 | 171.13 | 42.44 | 152.08 | 88.97 | 5.20 | 1155.04 | 17.92 |
| p-value | 0.18 | 0.21 | 0.34 | 0.41 | 0.36 | 0.65 | 0.52 | 0.68 | 0.042 | 0.049 |
| Mental excitement/ dispute | | | | | | | | | | |
| Yes | 27.54 | 71.28 | 116.87 | 168.66 | 39.70 | 151.45 | 94.25 | 3.80 | 688.41 | 12.95 |
| No | 27.08 | 72.49 | 116.28 | 172.53 | 42.52 | 149.69 | 92.31 | 5.40 | 1078.75 | 16.98 |
| p-value | 0.25 | 0.26 | 0.74 | 0.33 | 0.030 | 0.85 | 0.91 | 0.22 | 0.16 | 0.25 |
| Sleep disorder | | | | | | | | | | |
| Yes | 27.43 | 72.11 | 116.59 | 172.80 | 42.16 | 148.75 | 91.07 | 4.66 | 947.57 | 15.32 |
| No | 27.10 | 72.36 | 116.17 | 172.80 | 41.82 | 151.42 | 90.56 | 5.21 | 1015.76 | 16.51 |
| p-value | 0.28 | 0.78 | 0.77 | 1.00 | 0.74 | 0.71 | 0.97 | 0.59 | 0.77 | 0.67 |
| Changes at workplace | | | | | | | | | | |
| Yes | 26.96 | 70.17 | 116.18 | 174.56 | 41.51 | 161.30 | 83.48 | 4.87 | 771.62 | 12.77 |
| No | 27.21 | 72.77 | 116.38 | 172.75 | 42.05 | 149.38 | 92.94 | 5.14 | 1041.12 | 16.81 |
| p-value | 0.65 | 0.094 | 0.94 | 0.75 | 0.77 | 0.36 | 0.70 | 0.88 | 0.49 | 0.42 |
| Changes in partner relationship | | | | | | | | | | |
| Yes | 27.93 | 70.27 | 111.49 | 167.02 | 42.27 | 170.35 | 133.04 | 5.81 | 712.15 | 12.45 |
| No | 27.15 | 72.47 | 116.69 | 172.68 | 42.03 | 150.35 | 89.21 | 5.08 | 1020.71 | 16.44 |
| p-value | 0.32 | 0.33 | 0.15 | 0.48 | 0.93 | 0.27 | 0.19 | 0.78 | 0.58 | 0.58 |

[*] Body mass index  [#] Diastolic resting blood pressure  [§] Systolic resting blood pressure
[$] Cotinine level in serum  [&] C-reactive protein  [+] NT-pro BNP
